# Hospital Saturday in London

**Published:** 1895-09-21

**Authors:** 


### HOSPITAL SATURDAY IN LONDON.

The Hospital Saturday Fund is issuing an appeal for increased support, with a statement of the help it has afforded to hospitals and charitable institutions since its inception, 21 years ago. During last year 1,160 persons were sent to convalescent homes through the instrumentality of the Fund, and 2,296 surgical appliances were supplied, either altogether free or partially so. A useful ambulance service has recently been established for the benefit of riverside workers in South London, for whom, in cases of injury, no adequate provision existed for conveyance to the nearest hospital. The Street Ambulance Service of the Hospitals' Association has already provided for the removal of those who suffer injury in the streets of London. The Hospital Saturday Council appeals to those who know of workshops in which nothing is done in support of the hospitals to do their utmost to induce such firms to take up the matter, and promote the work of the Fund. As it is desired to publish the results of this appeal in the annual report, all contributions should be forwarded to the secretary, Mr. W. G. Bunn, 59, Farringdon Road, E.C., before December 31st, 1895.

